# Fluidized Bed Jet Milling Process Optimized for Mass and Particle Size with a Fuzzy Logic Approach

**DOI:** 10.3390/ma13153303

**Published:** 2020-07-24

**Authors:** Jaroslaw Krzywanski, Dariusz Urbaniak, Henryk Otwinowski, Tomasz Wylecial, Marcin Sosnowski

**Affiliations:** 1 Faculty of Science and Technology, Jan Dlugosz University in Czestochowa, Armii Krajowej 13/15, 42-200 Czestochowa, Poland; m.sosnowski@ujd.edu.pl; 2 Faculty of Mechanical Engineering and Computer Science, Czestochowa University of Technology, Armii Krajowej 21, 42-201 Czestochowa, Poland; urbaniak@imc.pcz.czest.pl (D.U.); otwinowski@imc.pcz.pl (H.O.); 3 Faculty of Production Engineering and Materials Technology, Czestochowa University of Technology, Armii Krajowej 19, 42-201 Czestochowa, Poland; wylecial@wip.pcz.pl

**Keywords:** fluidized bed jet milling technology, grinding, comminution, fuzzy logic, knowledge-based systems, simulation

## Abstract

The milling process is a complex phenomenon dependent on various technological and material parameters. The development of a fluidized bed jet milling model is of high practical significance, since milling is utilized in many industries, and its complexity is still not sufficiently recognized. Therefore, this research aims to optimize fluidized bed jet milling with the use of fuzzy logic (FL) based approach as one of the primary artificial intelligence (AI) methods. The developed fuzzy logic model (FLMill) of the investigated process allows it to be described as a non-iterative procedure, over a wide range of operating conditions. Working air pressure, rotational speed of the classifier rotor, and time of conducting the test are considered as inputs, while mass and mean Sauter diameter of the product are defined as outputs. Several triangular and constant linguistic terms are used in the developed FLMill model, which was validated against the experimental data. The optimum working air pressure and the test’s conducting time are 500 kPa and 3000 s, respectively. The optimum rotational speed of the classifier is equal to 50 s^−1^, considering the mass of the grinding product, and 250 s^−1^ for the mean Sauter diameter of the product. Such operating parameters allow obtaining 243.3 g of grinding product with the mean Sauter diameter of 11 µm. The research proved that the use of fuzzy logic modeling as a computer-based technique of solving mechanical engineering problems allows effective optimization of the fluidized bed jet milling process.

## 1. Introduction

Raw materials, as well as energy, are necessary to meet human needs. The growing demand for used materials leads to depletion of their resources. Furthermore, the potential of non-renewable energy resources, which dominate the global energy production structure, decreases every year. Moreover, the challenges of climate protection make it necessary to change the structure of world fuel. Raw materials and energy are becoming more expensive and more difficult in the context of their acquisition. The above circumstances have caused a change in the technology model for the production of raw materials and energy i.e., from a linear economy to a closed-cycle economy. This change of the paradigm in manufacturing technology is essential for both economic and ecological reasons.

Linear producing methods necessary in meeting the growing needs of humanity have resulted in a diminishing rate of both raw materials and energy. The closed-cycle economy concept is characterized by, among others, the fact that the smallest amount of fresh raw material is used in the delivering new products, while the product in the phase when its usefulness ends, is used for recovery and reuse. The same applies to the propriety of energy used in the production process. Used products are assessed in the context of energy recovery.

Those elements of a used product that cannot be sent back to production are directed to such methods of utilization that they do not constitute post-production waste and to recover energy previously necessary for their production.

Saving raw materials and energy is another element of the closed-cycle economy, which leads to optimization of manufacturing processes.

In many technologies, granular materials are used to manufacture finished products. Appropriate granularity of these materials is most often the result of a previously carried out grinding, which very often is a high energy consumption process. In the context of the closed-cycle economy, it is, therefore, crucial to carry out the process in a strict energy and material saving regime.

A grinding process is a complex phenomenon as many process occur simultaneously [1,2,3]. It depends on many parameters, such as different physical and chemical properties of the material being ground: homogeneity, strength, hardness, humidity, viscosity, forces and stresses occurring in said particles, internal particle structure, material defects of ground particles, and mill construction. A random occurrence often characterizes these variables [1,4,5,6].

Of the various grinding methods, pneumatic comminution has several advantages. One of the essential benefits of this grinding method is purity of the final product [2]. The use of air as a working medium in the comminution process results in particle size reduction without inclusions of foreign substances in the grinding product. If a different method of grinding is used, not pneumatic, the use of other materials to supply energy for grinding causes wear of these bodies (the formation of fine particles) and thus the presence of impurities in the final grinding product.

Currently, the most commonly used types of pneumatic mills are fluidized bed jet mills. They combine the advantages of jet milling and the intensity of particle movement in the fluidized bed, leading to a significant increase in process efficiency.

Since the complexity of the phenomena occurring in these types of mills does not allow one to obtain a clear description of the grinding process, experimental and model studies are interesting, timely, and of high practical significance. In the case of a grinding process, the primary purpose of the research is to describe a product. This description concerns two aspects. One is the amount of material produced as a result of the process, and the second aspect is the particle size distribution of the product [3]. 

On the other hand, the amount of product describes the efficiency of the process and, at the same time, its energy consumption. It also allows one to specify the cost of grinding. The second aspect is essential from the potential of further application of the product. The presence of coarse particles in the product may prevent its further use in many technological processes and there are several technologies where grinding products contain excessively fine particles which cannot be used. 

Several papers deal with fluidized bed jet mills. Article [4] presented the results of experimental research of a fluidized bed jet mill. The study aimed to describe the dynamics of complex two-phase processes taking place in the fluidized bed. The influence of particle size distribution and amount of feed mass, as well as the concentration of solid phase in the air on the efficiency of grinding glass balls, was investigated. On the basis of the experimental data, a simple Kapur’s model for batch grinding was applied. The authors in [5] described changes in the structural and functional properties of starch after the jet milling process. The results of jet milling efficiency of various inorganic materials (white sand, glass, iron oxide, hard coal, potassium and aluminum) with a narrow particle size distribution were considered in [6]. As shown, the authors managed to obtain a product below 1 µm with a narrow particle size distribution. Theoretical and experimental study of the kinetics of tire grinding was discussed in [7]. For this purpose, population balance modeling was performed. The study of tungsten powder comminution in a fluidized bed jet mill was shown in [8]. The authors demonstrated that the use of a fluidized bed jet mill allowed obtaining a product with improved effective particle dispersion. Besides, the use of a fluidized bed for grinding tungsten powder created conditions for correct (sharp) classification of the solids. Similar results were reported for grinding the TiAl alloy with the addition of Nb [9]. 

An interesting dynamic model of the closed loop grinding plant was developed by Gommeren et al. [2]. The authors observed that particle transport and size reduction inside the mill show a stochastic behavior and are described by size and state dependent probability functions. In spite of the fact that the model is two-dimensional and the tangential and radial velocity components are not functions of place, hold-up and supplied air produce realistic results for the stable operating range of the mill.

The theoretical analysis and results of experimental investigations of the fluidized bed jet milling process of silicon carbide were depicted in [10].

The influence of the grinding chamber nozzle size, process parameters and particle size of the feed on the shape of the product particles was shown. Model of the grinding and classification processes in the fluidized bed jet mill based on the Markov chains approach was proposed, and experimental research of the combined grinding and classification operations was carried out in [11]. The entropy model of the comminution and matrix model of multi-stage classification was presented in [12] and [13], respectively. The kinetics of grinding and classification of a mixture of granular materials with different grindability in fluidized bed jet mill was shown in [14]. The authors proposed a model based on a discrete analog of the Boltzmann equation capable of describing the kinetics of such a grinding process in a fluidized bed jet mill. 

A complex ANFIS model was developed to assess life and energy cycles of white rice in milling factories of Guilan province in Iran. The implemented multi-level ANFIS approach revealed that two-level ANFIS architecture comprising of three ANFIS models in the first level and one ultimate model in the third level provided the best forecasting performance for output energy, economic profit and GWP for converting paddy to white rice [15].

The discrete element method (DEM), used to investigate the effects of grinding medium properties on particle flow and grinding performance in a simplified IsaMill was described in [16]. An interesting CFD model of a jet mill was also developed in [17]. The authors observed that the proposed jet mill was advantageous for obtaining a pulverized product having a narrower particle size distribution. Applications of sometimes sophisticated strategies used by these methods for dividing the mill into a series of cells were depicted in detail in [16,18,19].

The above literature review showed that besides laborious and expensive experiments, the models, sometimes complicated and time-consuming, were the main techniques used to study the grinding processes. The fuzzy logic (FL) approach constitutes an alternative method of data handling [20,21,22]. It belongs to artificial intelligence (AI) techniques, allowing building intuitive and efficient models. Linguistic terms are main concepts used to describe the input and the output data in an FL model. The input signals’ processing demands the input parameters to be divided into fuzzy sets, where a numeric value of a considered parameter is assigned to a membership function value ranging from 0 to 1. Fuzzy rule base defines relationships between inputs and outputs [20,23].

Fuzzy logic methods are widely used in various engineering applications. Some authors [24] explored the use of fuzzy models for the Hot-Rolling industrial nonlinear process. They developed three fuzzy models for the rolling force, torque, and slab temperature. The models were successfully validated against the experimental data. 

The application of a fuzzy logic-based computer model of hydrocyclone was demonstrated in [25]. The authors underlined that the developed fuzzy model of a hydrocyclone could be used to simulate hydrocyclone circuits as these models can overcome the shortcomings of more traditional ones in the description of the performance of hydrocyclone circuits.

A multi-criteria fuzzy logic methodology aimed to perform impact category weighting and sensitivity analysis within the life cycle assessment of products and processes. The authors proved that the fuzzy interference system provides a novel approach to modeling the weighting factors using fuzzy membership functions [26]. 

A generic scheme to establish the norm for automation of design by employing fuzzy logic and neural networks for a surface grinding process was proposed in [27]. A fuzzy inference system was used to predict the amount of tool wear as a function of spindle speed, feed rate and measured machining forces in end milling of glass fiber reinforced polymer composites [28]. The results of end milling experiments showed that the fuzzy logic model was capable of estimating tool wear with excellent accuracy in the highly nonlinear region of tool wear and the machining forces relationships. A fuzzy inference model for prediction of tool life during end milling of the composite material was developed in [29]. Statistical analyses and validation experiments confirmed the accuracy of the model. An attractive fuzzy logic-based model to predict surface roughness of a machined surface in glass milling operation using a cubic boron nitride grinding tool was also implemented in [30]. The results demonstrated a settlement between the fuzzy model and experimental results with above 93% accuracy. The observations reported in [31] indicated that the fuzzy logic modeling method could also be effectively used for the prediction of surface roughness in face milling. 

Several fuzzy logic models for selecting machining parameters (cutting speed and feed rate) in drilling and milling type machining operations were presented in [32]. The results showed that the fuzzy logic approach could be advantageously used in the building of new generation automated process planning systems. Fuzzy logic techniques were also employed in the modeling of comminution processes. It was found experimentally that developed a feedback control system with fuzzy logic was a useful method for the control of product particle size during the dry grinding process in hammer mill [33]. A fuzzy controller was designed for the control flow rate inside the ball mill to avoid overfilling or emptying the mill [34]. The fuzzy system was used to optimize the ball milling circuit [35]. Nonlinear dynamics of the cement ball mill system was approximated using the fuzzy logic approach in [36]. The simulation results were given for different operating conditions. A method of fuzzy internal model control was successfully used for the grinding-classification system [37]. Fuzzy logic control systems used in the mining industry allowed for effective operational performance of mineral processing grinding circuits [38]. 

Summarizing, one should underline that besides laborious, time-consuming and expensive experiments, different models for simulation of jet milling processing can be found. However, models require some additional data (e.g., to adjust parameters) and, in some cases, are time-consuming when a desired accuracy need to be reached. The algorithms are often complicated and adjusting their parameters is not determined immediately, especially for different operating conditions.

The above literature review reveals that the latest bibliography lacks fuzzy logic-based models of such complex comminution processes as in fluidized bed jet mills, even though these mills are widely used in chemical, pharmaceutical, electrotechnical, food, and other industries. The fuzzy logic approach may be regarded as a useful optimization method. The possibility of obtaining results in a short time and their high accuracy are its primary advantages.

To the best of our knowledge, the present work is the first in the field literature dealing with the application of the FL methods for modeling grinding processes in fluidized bed jet mills. Grinding product mass and its mean Sauter diameter, over a wide range of operating conditions, was successfully predicted by the developed FLMill model. The necessary samples are taken from the experiments, similar to the methods described in [23,39,40].

## 2. Materials and Methods 

### 2.1. Subject of Research

In the case of fluidized bed jet grinding, the required product is obtained as a result of collisions of the ground particles, as well as their mutual abrasion [12,13,41,42]. 

It is possible as the solids inside the bed behave like liquid. Since fluidized gas is introduced at high pressure through the bed, the solids are lifted and suspended in a stream of gas. Due to rigorous mixing in fluidized beds, the gas bubble size, shape, formation, rising velocity and coalescence in the fluidized beds have quantitative similarity with those of gas bubbles in liquids, improving solid and fluid contacting [43]. Thus, collisions of the ground particles and their abrasion depend on the energy of the particles and the probability of a collision or abrasion influenced by the concentration of particles in the grinding chamber. Therefore, the final effect of the process, measured by the particle size distribution of the grinding product and the efficiency of the process depend, among others, on the initial particle size and feed mass, the pressure and temperature of the working air, the rotational speed of the classifier rotor and the duration of the grinding test. 

**Figure 1:**
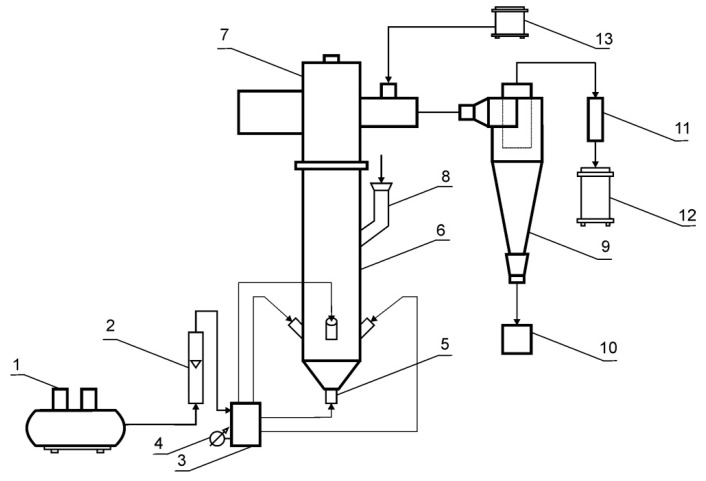
Schematic diagram of the experimental set-up; 1—compressor, 2—rotameter, 3—working air collector, 4—elastic pressure gauge, 5—air nozzles, 6—grinding chamber, 7—rotational flow classifier with the electric motor, 8—feed material filling container, 9—cyclone, 10—grinding product I box, 11—cloth filter (grinding product II), 12—vacuum for exhaust air, 13—vacuum for seal air.

The fluidized bed jet mill cooperates with a classifier. In the case of the mill system being the subject of the research, a centrifugal flow classifier was used whose operation depends primarily on the rotational speed of the rotor. The higher the speed values, the smaller the particle going to the grinding product zone. Therefore, experimental studies were carried out, the aim of which was to determine the influence of the basic parameters of the grinding process on its performance. The investigation of the comminution was conducted in a laboratory fluidized bed jet mill schematically presented in Figure 1.

**Figure 2:**
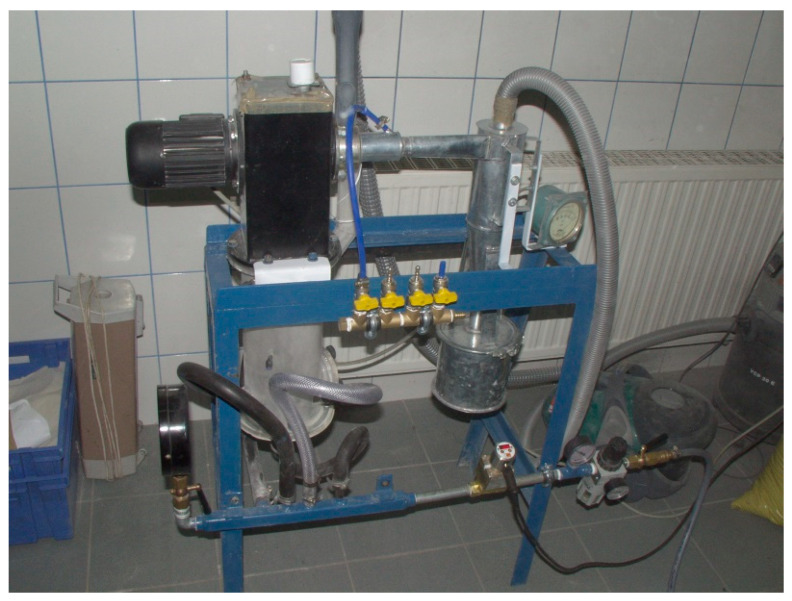
View of the testing stand.

The working air is supplied to the mill by a compressor (1), and the sealing air is supplied by a vacuum pump (13). The granular material is fed to the cylindrical grinding chamber (6) gravitationally from the charging container (8). The fed material undergoes extensive fluidization employing air jets from the nozzles (5). The nozzles inject air concentrically at a controlled flow rate and overpressure p with flow velocity u. The mass flow rate of the working air is measured by the rotameter (2), while the overpressure is measured by the elastic pressure gauge (4). The rotor classifier (7) is installed above the grinding chamber (6) in the range of fountain flow of ground particles. The classifier separates the ground material into the fine and coarse fraction directed to the cyclone (9), and the grinding chamber for repeated grinding. The cut size of classification can be controlled by regulating the angular speed of the rotor. Separation of the fine particles from the air after classification occurs in the cyclone. The air-particle mixture is separated into partially dedusted working air that is directed to the cloth filter (11) and the flow of grinding product I that is guided to the container (10). The solid particles in the air, which were not captured in the cyclone, are caught by a fabric filter. These particles, as grinding product I, together with grinding product II, constitute the total product of fluidized bed jet milling. Negative pressure is assured by a vacuum (12). A view of the testing stand is presented in Figure 2.

The experimental tests in the fluidized bed jet mill were carried out in a batch mode. This type of grinding is rare in the industry, but the purpose of the research was to check the results of the numerical simulation of the process. Samples of limestone of a particle size ranging from 800 to 1200 µm, which had previously been selected using a sieve set, were subjected to testing. The particle size distribution of limestone feed is shown in Table 1.

Limestone from the mine “Czatkowice” in Krzeszowice near Cracow, Poland, with a bulk density of 2680 kg/m^3^, was employed in the research. The weight of the feed was 1500 g. Comminuted substance from the grinding chamber and the feed were weighed on electronic lab scales with an accuracy of 0.01 g. The research was carried out with the following variable parameters: the value of the test conducting time, the working air pressure, and the classifier rotor speed. The experiments were carried out for five values of each parameter. The test conducting time and the working air pressure were measured with a stopwatch with an accuracy of 1 s and a pressure gauge with an accuracy of 10 kPa, respectively. The rotor speed of the classifier was determined using a tachometer with a precision of 0.1 s^−1^.

**Figure 3:**
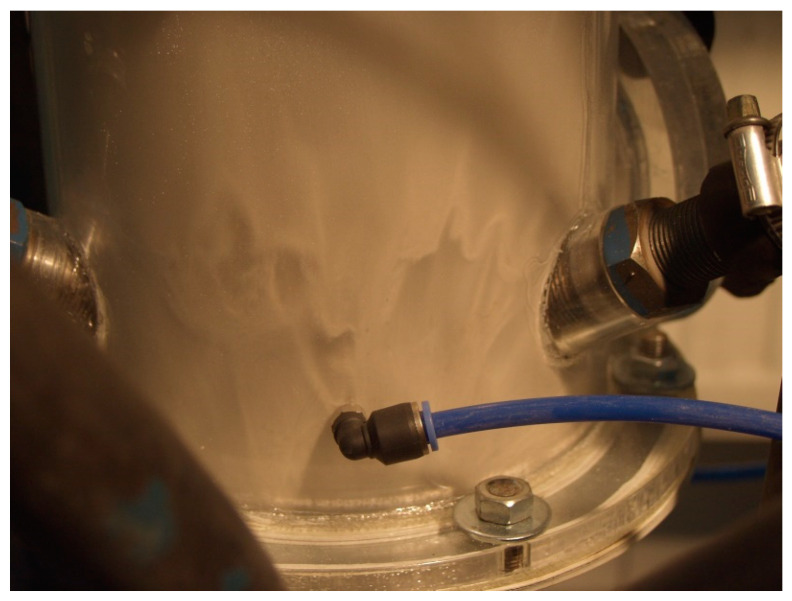
View of the fluidized bed in the mill chamber.

In all the grinding research, the following measurements were made: mass and particle size distribution of the feed and product from the grinding chamber, pressure, temperature and flux of the working air mass as well as environmental parameters. Figure 3 shows a sample picture of the fluidized bed during the experiments. The particle size distribution of the representative samples of the feed and product were measured by an Infrared Particle Sizer manufactured by Kamika Instruments [44].

### 2.2. Fuzzy Logic-Based Grinding Model

The fuzzy logic (FL) method was selected in the study of the complex fluidized-bed jet milling process as this technique is useful when subjective knowledge of an expert is significant in defining an objective function and decision variables [45]. The FL approach belongs to so-called artificial intelligence (AI) methods and is convenient for prediction and optimization tasks [20,45,46,47,48,49,50]. This technique allows building knowledge-based systems for solving complex problems [51]. The method is based on the use of fuzzy sets and linguistic variables to describe the behavior of a process or an object, dealing with imprecise, vague and uncertain information and employing qualitative judgment to parameters quantitative in nature [20,23,39,40]. Thus, the approach allows considering tolerance and imprecision in the description of a process and building more robust models of reality, including complex systems [52,53,54]. 

These features are essential due to the high complexity of the fluidized-bed jet milling mechanisms.

The fuzzy system consists of four stages, i.e., fuzzification, rule base, inference and defuzzification [55,56,57,58]. The FL approach uses membership functions μ, which assigns a numerical value of an input variable a certain value in the range [0, 1] called the grade of membership [20]. This procedure is the so-called fuzzification stage, providing fuzzy sets S, which can be expressed by Zadeh’s notation as follows [23,52,59]:

(1) $$S=\left\{\mu_{S}\left(i_{1}\right)/i_{1}+\mu_{S}\left(i_{2}\right)/i_{2}+\ldots +\mu_{S}\left(i_{n}\right)/i_{n}\right\},$$

where *μ_s_* is the membership functions of fuzzy set *S, i_1_, i_2_, …i_n_*—input variables.

Thus, the fuzzification block calculates the grade of membership of each crisp value to individual fuzzy sets. Different membership functions can be used when developing an FL model: triangular, trapezoidal or gaussian [20,45,59,60,61]. 

Based on the determined input grades of membership, the resulting value of the membership function of the model output is calculated in the inference stage. However, the rule base is necessary to conduct the inference calculations. The rule base contains logical IF-THEN rules, defining the cause and effect relationships existing in the system between fuzzy sets of inputs and outputs and representing linguistic reasoning [45]. Thus, the IF-THEN fuzzy rule base allows the fuzzy outputs *O* to be generated. Two main methods of deductive inference for fuzzy systems can be distinguished: the Mamdani and Sugeno techniques. In Mamdani models a fuzzy system with two inputs (antecedents) *i_1_* and *i_2_* and a single output (consequent) o can be described by the set of linguistic IF-THEN rules in the form:

IF *i_1_* is *S_1_* and *i_2_* is *S_2_* THEN *o_1_* is *O*, (2)

where output *O* is a fuzzy set.

A typical fuzzy rule with two inputs *i_1_* and *i_2_* and two outputs o_1_ and *o_2_* with the Takagi–Sugeno engine [62], sometimes also called the TSK models (Takagi, Sugeno, and Kang), can be written as follows:

IF *i_1_* is *S_1_* and *i_2_* is *S_2_* THEN o_1_ is *O_1_* = f_1_(*i_1_*, *i_2_*) and *o_2_* is *O_2_* = f_2_ (*i_1_*, *i_2_*), (3)

where f_1_ (*i_1_*, *i_2_*) and f_2_ (*i_1_*, *i_2_*) are polynomial functions of inputs *i_1_* and *i_2_*.

The Takagi–Sugeno approach is widely used due to its flexibility and ease of use [45]. 

Finally, during the defuzzification stage, based on the resulting grade value of the membership function of the output, the crisp output value is calculated as the result of crisp inputs. An essential feature of the method is the fact that it allows a practical problem to be formalized using experience rather than strict knowledge of the process, and the theory is not essential here [20,39]. A detailed description of the method can be found elsewhere [20,55,56,57].

Validation of the developed FLMill model was successfully performed against the experimental results, i.e., the mass of the product and the mean Sauter diameter of the product. The QtFuzzyLite fuzzy logic control application was employed during the study [63]. Qtfuzzylite software (version 5.0b1408, FuzzyLite Limited, Wellington, New Zealand) was used to develop the model. In the authors’ opinion, this software is a more intuitive and flexible tool than the MATLAB, although the use of the FL toolbox of MATLAB is also possible in the considered case. However, simplicity in use, i.e., friendly to use the graphical user interface (GUI) and the intuitiveness of Qtfuzzylite, convinced the authors to choose this tool in this study. The presented approach allowed for obtaining results immediately, so fast computations are the main advantages of the performed FLMill model.

The following three input parameters are assumed in the model: pressure p of the working air, rotational speed n of the classifier and time t of conducting the test. Mass m of the product and mean Sauter diameter d of the grinding product constitute the output parameters. The inputs and outputs are described in Table 2.

**Figure 4:**
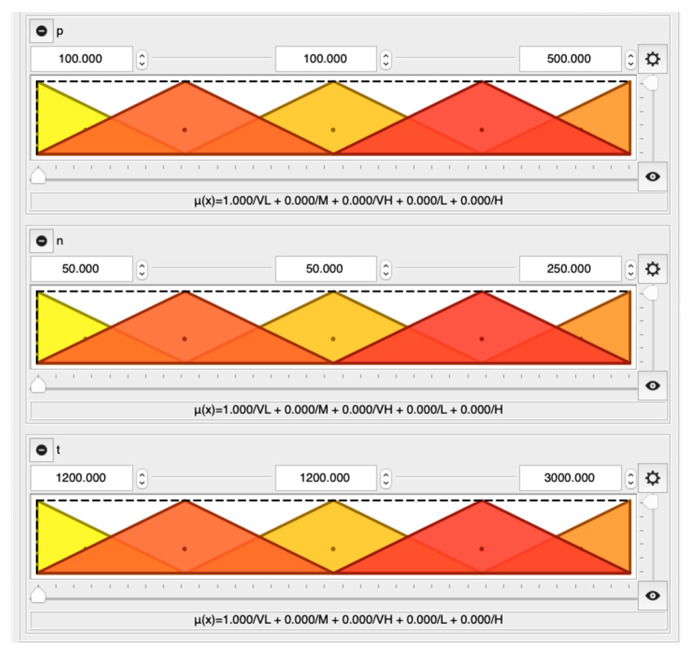
Membership functions for input parameters: p, n, t (x-axes and y-axes correspond to parameter values from Table 2 and values of membership function, respectively).

These three input parameters i.e., p, n, t, are described by five overlapping triangular linguistic terms: very low (VL), low (L), medium (M), high (H), very high (VH), shown in Figure 4. Despite the fact, that various types of linguistic terms are available in QtFuzzyLite application, e.g., triangular, trapezoidal, rectangular, discrete, gaussian, bell, cosine, pi shaped, sigmoidal, s-shaped, z-shaped, constant and linear, the most commonly used triangular linguistic terms are employed in this study (Figure 4).

**Figure 5:**
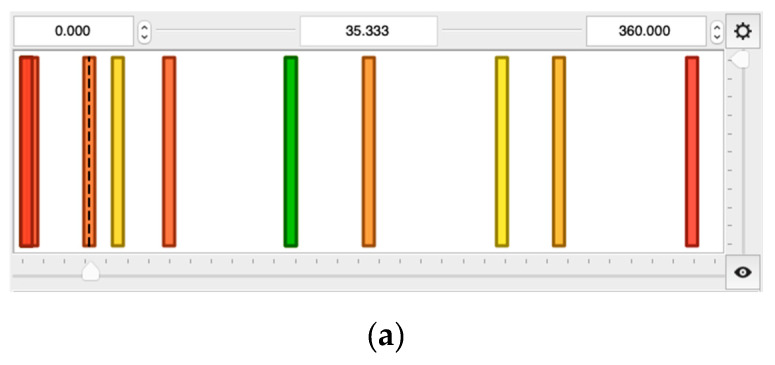

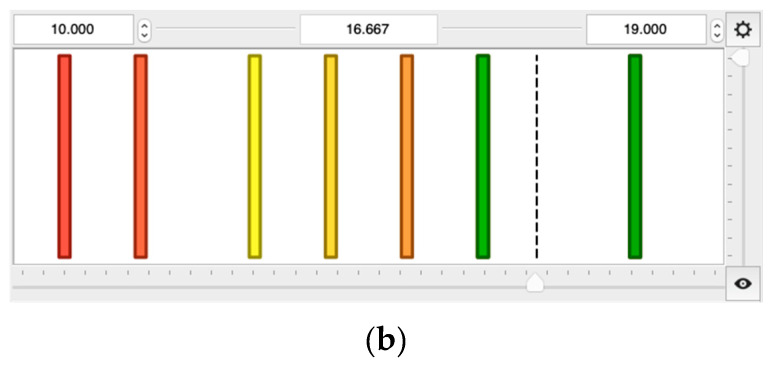
Membership functions for output parameters; x-axes correspond to the mass of the grinding product (**a**) and mean Sauter diameter of the product (**b**) from Table 2, whereas y-axes express membership function value.

Two outputs, i.e., mass m and the mean Sauter diameter d of the grinding product, were considered in the model. To fully and accurately describe them, five constant linguistic terms, i.e., very low (VL), low (L), medium (M), high (H), very high (VH), are used as depicted in Figure 5. Such arranged inputs and outputs can express the influence of each input on the outputs. Thus, during the fuzzification step, for the considered application, vectors of crisp inputs are transformed into vectors of membership degrees i.e., fuzzy sets expressed by the Zadeh’s formula:

(4) $$S=\left\{{µ}_{S}\left(VL\right)/VL+{µ}_{S}\left(L\right)/L+{µ}_{S}\left(M\right)/M+{µ}_{S}\left(H\right)/H+{µ}_{S}\left(VH\right)/VH\right\},$$

The set of fuzzy rules for mass m and mean Sauter diameter d of the product are formulated according to Table 3 and Table 4.

For this work, the Takagi–Sugeno inference engine is used to determine the fuzzy output variable [20]. The weighted average method is employed during the defuzzification stage to establish a crisp output value in the defuzzification stage as it is one of the most common and computationally efficient methods [20]:

(5) o=∑µS (o¯)·o¯∑µS (o¯),

where $\bar{o}$ is the centroid of each membership function.

The developed FLMill model may be regarded as a useful optimization tool. The possibility of obtaining results in a short time and their high accuracy are the primary advantages of the method. On the other hand, the availability of expert knowledge and a small number of input variables are the main limitations of this technique. Intelligent hybrid systems, combining fuzzy logic, neural networks, and genetic algorithms, may overcome these shortcomings.

## 3. Results and Discussion

**Figure 6:**
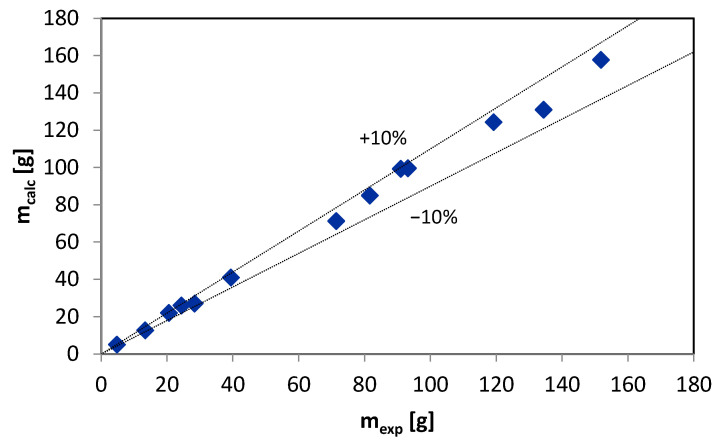
Comparison of measured and predicted by FLMill model mass of the grinding product.

**Figure 7:**
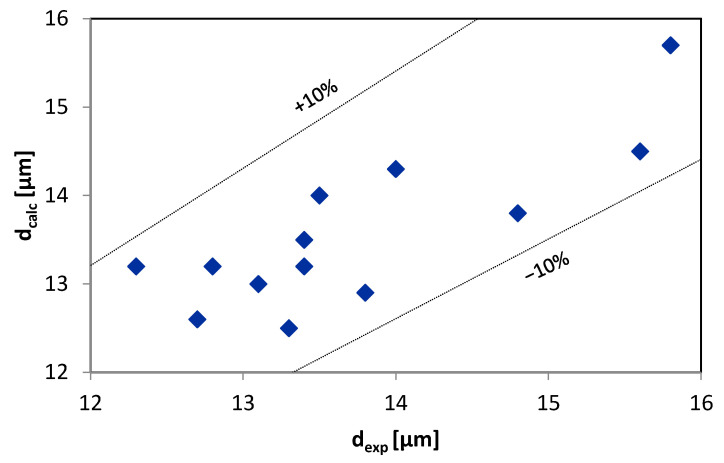
Comparison of measured and predicted by the FLMill model mean Sauter diameter of grinding product.

A comparison between the measured data and the data calculated by the FLMill model is shown in Figure 6 and Figure 7. The calculated results are located within the range of ±10% for mass and mean Sauter diameter of the product, compared to the experimental results. The data shown in Figure 6 and Figure 7 correspond to different values of input parameters as the comparison between the desired and calculated data is regarded as the most demanding and challenging type of model validation procedure [64,65,66,67]. 

A similar comparison is also given in Appendix A. Most of the errors are lower than 5%. The higher errors may be the result of difficulties in proper assessment of mass m of the product generated during the grinding process as the whole grinding process is complex, and some amounts of the material tend to adhere and aggregated on different parts of the entire crushing facility and installation. Based on the data contained in Appendix A (Table A1 and Table A2), calculations of sums of the squared errors (SSE) of the prediction were performed. The sums SSE are used as a measure of fit quality. The calculated sum values indicate a better fit of the model to the experimental data of the Sauter diameter than the mass of grinding product, which confirms the observations mentioned above.

**Figure 8:**
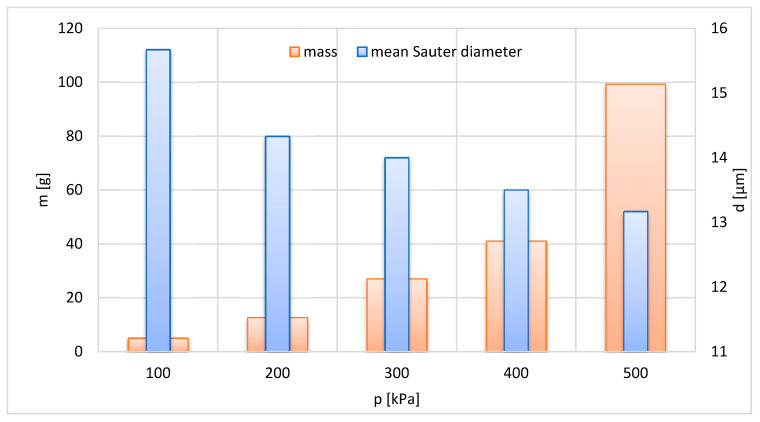
Effect of the pressure of working air on mass and mean Sauter diameter of grinding product; t = 1200 s, n = 150 s^−1^.

**Figure 9:**
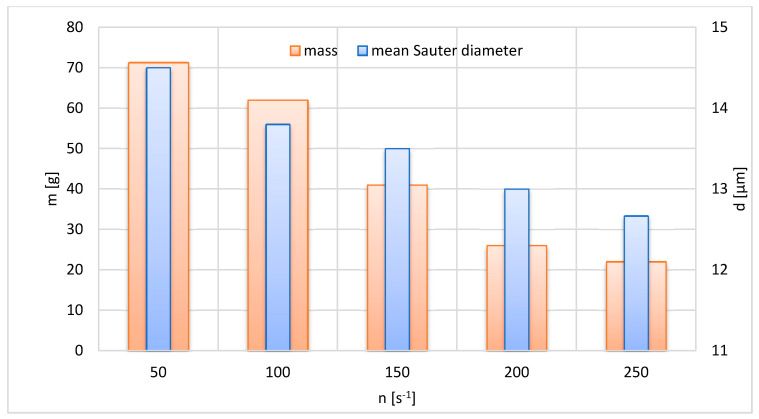
Effect of the rotational speed of the classifier rotor on mass and mean Sauter diameter of the grinding product; p = 400 kPa, t = 1200 s.

**Figure 10:**
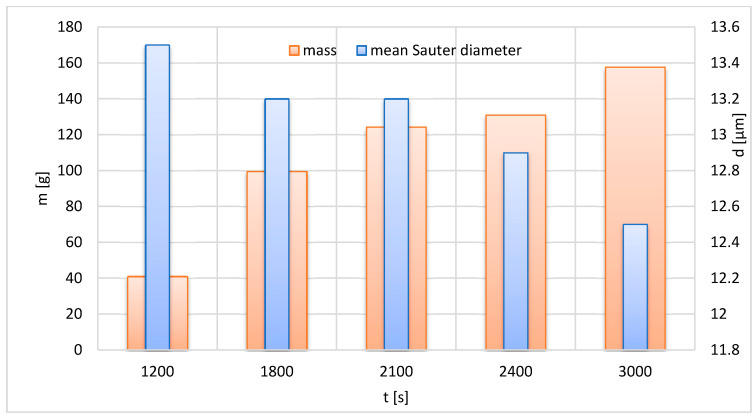
Effect of test conducting time on mass and mean Sauter diameter of the grinding product; p = 400 kPa, n = 150 s^−1^.

The developed FLMill model can be used as a tool to study the influence of the operating parameters on mass m of the grinding product and mean Sauter diameter d of the grinding product. One only needs to enter the input parameters and run the developed FLMill model. For the study of the influence of a specific operating input variable on the outputs, other inputs should be fixed as the dependence can be established only for the specified conditions [39] (Figure 8, Figure 9 and Figure 10).

The effect of working air pressure on the grinding performance is given in Figure 8 and Figure 10. The mass of the grinding product increases with the working air pressure. It is a result of an increase in energy of the working air with pressure. Therefore, the increase in p leads to a rise in mass m of the grinding product (Figure 8). The increased energy of the working air results in the increase in grinding efficiency leading to a decrease in the mean Sauter diameter d of the grinding product, according to results depicted in Figure 8. 

The influence of the rotational speed of the classifier n on the mass of the grinding product m and mean Sauter diameter d of the grinding product is shown in Figure 9. Since the increase in the angular speed of the rotor in the classifier improves the separation efficiency of the classifier, the increase in n causes a decrease in both the mass and mean Sauter diameter of the product. 

Figure 10 depicts the influence of conducting time of the test on mass and mean Sauter diameter of the grinding product. An increase in the duration of the test leads to an increase in the produced mass m. On the other hand, an increase in the duration of the grinding process leads to a decrease in the mean Sauter diameter of the obtained product. This behavior can be explained as follows. For more extended periods of conducting the test, the total energy supplied to the system consumed for the grinding process is higher. Thus, more extended periods of the grinding process result in a finer product produced in a fluidized bed jet milling mechanism. 

**Figure 11:**
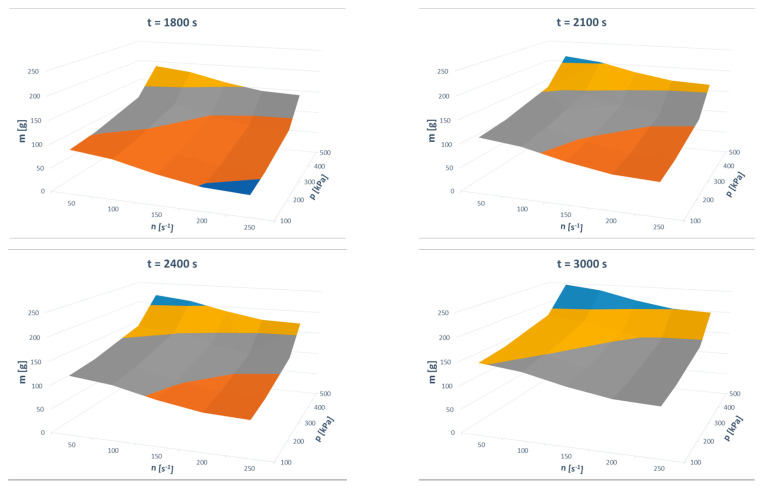
Effects of input parameters on the mass of grinding product.

**Figure 12:**
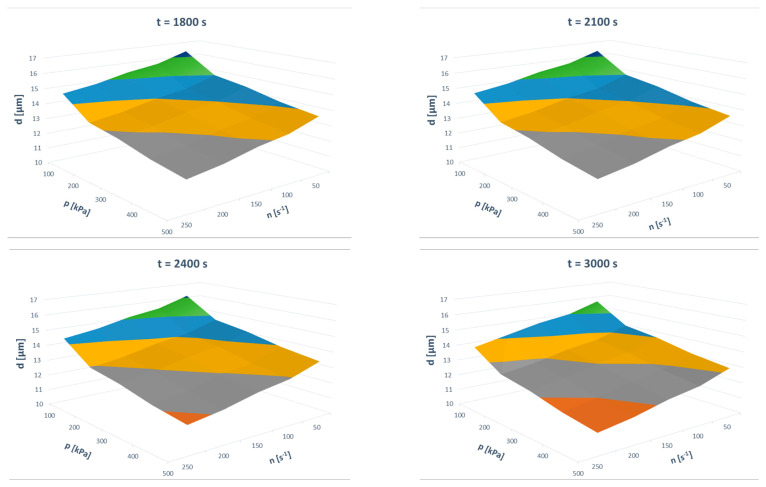
Effects of the input parameters on the mean Sauter diameter of the grinding product.

The model’s predictions can be easily presented in 3D plots. Such presentations are given in Figure 11 and Figure 12. These figures describe the impact of the three-dimensional input variable space on the two outputs of the model.

The developed FLMill model can serve as a useful simulation tool. It helps match the best-operating conditions corresponding to the grinding product of desired characteristics. The technique constitutes an alternative approach to other methods of data handling, considering the complexity of numerical and analytical methods as well as the high costs of time-consuming empirical experiments.

## 4. The Best Strategy for Performance of Fluidized Bed Jet Milling

Such established dependencies allow determination of the best strategy for performance of the fluidized bed jet milling process. The evaluation carried out in this study showed that the mass of grinding products, as well as its mean Sauter diameter of particles, depend on working air pressure (p), rotational speed of the classifier rotor (n) and time of conducting tests (t). 

The developed FLMill model allows selecting the optimal operational parameters, i.e., the highest grinding mass (m) and the lowest mean Sauter diameter (d) of particles. The selection of such parameters significantly increases the process efficiency making the technology effective, suitable for cleaner production and environmental conservation.

On the base of the observed trends, the effects of input parameters on the outputs can be summarized, as shown in Table 5.

As seen, the effectiveness of the fluidized bed jet milling process can be intensified by an increase in working air pressure and the test conducting time. The increase in rotational speed leads to a decrease in particles’ mean Sauter diameter, but also the mass of the grinding product, lowering the effectiveness of the process. Therefore, for the considered range of input operational parameters (Table 2), the highest mass of the grinding product is equal to 243.3 g and may be achieved for p = 500 kPa, n = 50 s^−1^, and t = 3000 s. On the other hand, the lowest mean Souter diameter of the grinding product particles is 11 µm, which can be obtained for p = 500 kPa, n = 250 s^−1^, and t = 3000 s. In other words, for the considered range of inputs, the optimum values of working air pressure and the test conducting time are 500 kPa and 3000 s, respectively. The optimum rotational speed of the classifier rotor is equal to 50 s^−1^ taking into account the mass, while 250 s^−1^ considering the mean Sauter diameter of the product.

## 5. Conclusions

This paper is the first available in open literature dealing with the use of the fuzzy logic method in the modeling of fluidized bed jet milling as an effective technology, suitable for cleaner production and environmental conservation.

The developed FLMill model allows the mass and the mean Sauter diameter of the grinding product to be predicted for different operating conditions. 

It has been shown that the proposed approach gives quick and accurate results as an answer to the input data sets. The results evaluated using the developed model are in good agreement with relevant experimental data. The maximum relative errors are lower than 10%. 

For the considered range of input operational parameters, the optimum values of working air pressure and the test conducting time are 500 kPa and 3000 s, respectively. The optimum rotational speed of the classifier rotor is equal to 50 s^−1^ and 250 s^−1^, for the mass of the product and its mean Sauter diameter, respectively.

The highest mass of the grinding product and the lowest mean Souter diameter of the grinding product is 243.3 g and 11 µm, respectively.

The FLMill model constitutes a useful computer-based simulation tool capable of predicting desired product characteristics from the fluidized bed jet mill process over a wide range of operating conditions.

As mentioned in the introduction, in the aspect of the closed cycle economy, optimizing production processes of various branches of the economy is extremely important. It allows planning the course of a given process with maximum use of primary raw materials, as well as maintaining the minimum energy necessary for its implementation. This is part of the idea of sustainable development which is extremely important due to the growing challenges of today.

## Figures and Tables

**Table 1:** The particle size distribution of limestone feed.

No	The Average Size of the Particle Class Di, µm	F(D_i_), %
0	0	0.00
1	34	0.00
2	74	0.00
3	114	0.00
4	154	0.00
5	194	0.00
6	234	0.00
7	274	0.00
8	314	0.03
9	354	0.10
10	394	0.27
11	434	0.78
12	475	2.00
13	515	3.92
14	555	8.04
15	595	14.84
16	635	24.61
17	675	34.79
18	715	45.82
19	755	56.56
20	795	66.39
21	835	74.04
22	875	81.46
23	915	86.09
24	955	89.91
25	995	92.28
26	1035	94.83
27	1076	96.45
28	1116	97.58
29	1156	99.10
30	1196	99.38
31	1236	100.00

**Table 2:** Variables of the model.

Parameter	Values
**Inputs**	
Working air pressure p, kPa	100–500
Classifier rotor speed n, s^−1^	50–250
Test conducting time t, s	1200–3000
**Outputs**	
Mass of the product, g	24.4–151.8
Mean Sauter diameter d of the grinding product, µm	12.3–15.6

**Table 3:** Fuzzy rule base for the mass of grinding product.

ID	Rule
1	If p is VL then m is VL*
2	if p is L then m is L
3	if p is M then m is M
4	if p is H then m is H
5	if p is VH then m is VH
6	If n is VL then m is VH
7	if n is L then m is H
8	if n is M then m is M
9	if n is H then m is L
10	if n is VH then m is VL
11	If t is VL then m is VL
12	if t is L then m is L
13	if t is M then m is M
14	if t is H then m is H
15	if t is VH then m is VH

* VL—very low, L—low, M—medium, H—high, VH—very high.

**Table 4:** Fuzzy rule base for mean Sauter diameter of the grinding product.

ID	Rule
1	if p is VL then d is VH
2	if p is L then d is H
3	if p is M then d is M
4	if p is H then d is L
5	if p is VH then d is VL
6	if n is VL then d is VH
7	if n is L then d is H
8	if n is M then d is M
9	if n is H then d is L
10	if n is VH then d is VL
11	If t is VL then d is VH
12	if t is L then d is H
13	if t is M then d is M
14	if t is H then d is L
15	if t is VH then d is VL

* VL—very low, L—low, M—medium, H—high, VH—very high.

**Table 5:** Effect of an increase in input parameters on mass of the grinding product and the mean Sauter diameter of the grinding product from fluidized bed jet milling.

Parameter (Horizontal Axis)	Mass of the Product from the Grinding Chamber	Mean Sauter Diameter of the Grinding Product
Working air pressure p	Increase	Decrease
Classifier rotor speed n	Decrease	Decrease
Test conducting time t	Increase	Decrease

## Appendix A

**Table A1:** Mass of grinding product, measured and calculated by FLMill model (SSE = 208.49).

p kPa	n s^−1^	t s	m_exp_ g	m_calc_ g	Err %
400	150	1200	39.4	41.0	−4.1
400	150	1800	93.2	99.6	−6.9
400	150	2100	119.2	124.3	−4.3
400	150	2400	134.4	131.0	2.5
400	150	3000	151.8	157.7	−3.9
100	150	1200	4.8	5.0	−4.2
200	150	1200	13.4	12.7	5.2
300	150	1200	28.4	27.0	4.9
400	150	1200	39.4	41.0	−4.1
500	150	1200	91.0	99.3	−9.1
400	50	1200	71.4	71.3	0.1
400	100	1200	81.6	85.0	−4.2
400	150	1200	39.4	41.0	−4.1
400	200	1200	24.4	26.0	−6.6
400	250	1200	20.6	22.0	−6.8

**Table A2:** Mean Sauter diameter of the product, measured and calculated by FLMill model (SSE = 5.07).

p kPa	n s^−1^	t s	d_32__exp µm	d_32__calc µm	Err %
400	150	1200	13.4	13.5	−0.8
400	150	1800	13.4	13.2	1.5
400	150	2100	12.3	13.2	−7.3
400	150	2400	13.8	12.9	6.5
400	150	3000	13.3	12.5	6.0
100	150	1200	15.8	15.7	0.6
200	150	1200	14.0	14.3	−2.1
300	150	1200	13.5	14.0	−3.7
400	150	1200	13.4	13.5	−0.8
500	150	1200	12.8	13.2	−3.1
400	50	1200	15.6	14.5	7.1
400	100	1200	14.8	13.8	6.8
400	150	1200	13.4	13.5	−0.8
400	200	1200	13.1	13.0	0.8
400	250	1200	12.7	12.6	0.7
